# The effect of hyperbaric oxygen therapy on bone macroscopy, composition and biomechanical properties after ionizing radiation injury

**DOI:** 10.1186/s13014-020-01542-2

**Published:** 2020-05-06

**Authors:** Luiz Henrique Ferreira Júnior, Pedro Henrique Justino Oliveira Limirio, Priscilla Barbosa Ferreira Soares, Paula Dechichi, Letícia de Souza Castro Filice, Paulo Sérgio Quagliatto, Flaviana Soares Rocha

**Affiliations:** 1 Integrated Dental Clinic Program, Faculty of Dentistry, Federal University of Uberlândia, Avenida Pará s/n°, Campus Umuarama, Bloco 4L, Bairro Umuarama, Uberlândia, Minas Gerais 38.400-902 Brazil; 2 Department of Periodontology and Oral Implantology, Faculty of Dentistry, Federal University of Uberlândia, Avenida Pará s/nº, Campus Umuarama, Bloco 4L, Bairro Umuarama, Uberlândia, Minas Gerais 38.400-902 Brazil; 3 Department of Cell Biology, Histology and Embryology, Faculty of Dentistry, Federal University of Uberlândia, Avenida Pará s/nº, Campus Umuarama, Bloco 2B, Bairro Umuarama, Uberlândia, Minas Gerais 38.400-902 Brazil; 4 Department of Clinical Medicine, Histology and Embryology, Faculty of Medicine, Federal University of Uberlândia, Avenida Pará s/n°, Campus Umuarama, Bloco 4U, Bairro Umuarama, Uberlândia, Minas Gerais 38.400-902 Brazil; 5 Department of Dentistry and Dental Materials, Faculty of Dentistry, Federal University of Uberlândia, Avenida Pará s/nº, Campus Umuarama, Bloco 2B, Bairro Umuarama, Uberlândia, Minas Gerais 38.400-902 Brazil; 6 Department of Oral and Maxillofacial Surgery and Traumatology and Implantology, Faculty of Dentistry, Federal University of Uberlândia, Avenida Pará s/nº, Campus Umuarama, Bloco 2B, Bairro Umuarama, Uberlândia, Minas Gerais 38.400-902 Brazil

**Keywords:** Tibia, Ionizing, radiation, Hyperbaric oxygenation, Bone matrix, Osteogenesis, Biomechanical phenomena

## Abstract

**Background:**

Radiotherapy used in tumor treatment compromises vascularization of bone tissue. Hyperbaric oxygenation (HBO) increases oxygen availability and improves vascularization, minimizing the deleterious effects of ionizing radiation (IR). Therefore, the aim of this study was to evaluate HBO therapy effect on bone macroscopy, composition and biomechanical properties after IR damage.

**Methods:**

Twenty male *Wistar* rats weighing 300 ± 20 g (10 weeks of age) were submitted to IR (30 Gy) to the left leg, where the right leg was not irradiated. After 30 days, ten animals were submitted to HBO therapy, which was performed daily for 1 week at 250 kPa for 90-min sessions. All animals were euthanized 37 days after irradiation and the tibia were separated into four groups (*n* = 10): from animals without HBO - right tibia Non-irradiated (noIRnoHBO) and left tibia Irradiated (IRnoHBO); and from animals with HBO - right tibiae Non-irradiated (noIRHBO) and left tibia Irradiated (IRHBO). The length (proximal-distal) and thickness (anteroposterior and mediolateral) of the tibiae were measured. Biomechanical analysis evaluated flexural strength and stiffness. Attenuated Total Reflectance Fourier Transform Infrared Spectroscopy (ATR-FTIR) was used to calculate the amide I ratio, crystallinity index, and matrix to mineral ratios.

**Results:**

In the macroscopic and ATR-FTIR analysis, the IRnoHBO showed lower values of length, thickness and amide I ratio, crystallinity index and matrix to mineral ratios compared to noIRnoHBO (*p* < 0.03). IRnoHBO showed no statistical difference compared to IRHBO for these analyses (*p* > 0.05). Biomechanics analysis showed that the IRnoHBO group had lower values of flexural strength and stiffness compared to noIRnoHBO and IRHBO groups (*p* < 0.04). In addition, the noIRHBO group showed higher value of flexural strength when compared to noIRnoHBO and IRHBO groups (*p* < 0.02).

**Conclusions:**

The present study concluded that IR arrests bone development, decreases the collagen maturation and mineral deposition process, thus reducing the flexural strength and stiffness bone mechanical parameters. Moreover, HBO therapy minimizes deleterious effects of irradiation on flexural strength and the bone stiffness analysis.

## Introduction

Ionizing radiation (IR) used in radiotherapy treatment of patients with neoplastic lesions [1] induces hypovascularity, hypoxia and reduction of bone cells, impairing bone regenerative and remodelling [2, 3]. Studies showed that IR in the bone growth plate induces acute cell death, affects the proliferation/maturation pathway of chondrocytes [4] and arrests the bone growth process [5]. In addition, IR can stimulate water radiolysis, which causes collagen molecules denaturation at the interfacial bond with hydroxyapatite (HA) [6], compromising the mechanical properties, due to changes in the bone hierarchical arrangement [7].

Hyperbaric oxygen (HBO) therapy, performed in a chamber with 100% oxygen at a pressure between 200 and 250 kilopascal (kPa), may be used to treat bone damage by irradiation to improve bone metabolism. Inhalation oxygen at partial pressures over 200 kPa increases the production of reactive oxygen species, which act as signaling molecules for some growth factors, cytokines, and hormones [8]. In addition, study performed in diabetic animals showed that HBO therapy may contribute to incorporation mineral crystals into collagen crosslinks, increasing the maximum fracture strength [9]. In irradiated animals, HBO therapy has shown to increase bone regeneration in non-irradiated group and promote angiogenesis in irradiated group [10, 11]. However, the effects of HBO therapy on bone matrix properties, compromised by irradiation, still remains unknown.

Radiotherapy applied to patients with neoplastic lesions has been associated with abnormal bone structure development [12], increased risk of fracture [13] and mandibular osteoradionecrosis [14]. Thus, there is a need to study treatments to minimize these deleterious effects and improve the life quality of such patients. Therefore, the aim of the present study was to evaluate IR and HBO effects on the tibia of rats, using macroscopic, biomechanics and Attenuated Total Reflectance Fourier Transform Infrared Spectroscopy (ATR-FTIR) analyses.

## Material and methods

Twenty male Wistar rats (*Rattus norvergicus*) weighing 300 ± 20 g (10 weeks of age) were housed in standard conditions (12 h light/dark cycle, temperature of 22 ± 1 °C and relative humidity of 50–60%), with food (composition: humidity, crude protein, ethereal extract, mineral, crude fiber, calcium and phosphorus) and water ad libitum. All experimental protocols with animals were approved by the Committee on the Ethics of Animal Use and Care of the Federal University of Uberlândia (permit number 028/12). All procedures were carried out in strict accordance with the recommendations in the Guide for the National Institutes of Health guide for the care and use of Laboratory animals (NIH Publications No. 8023, revised 1978).

**Fig. 1:**
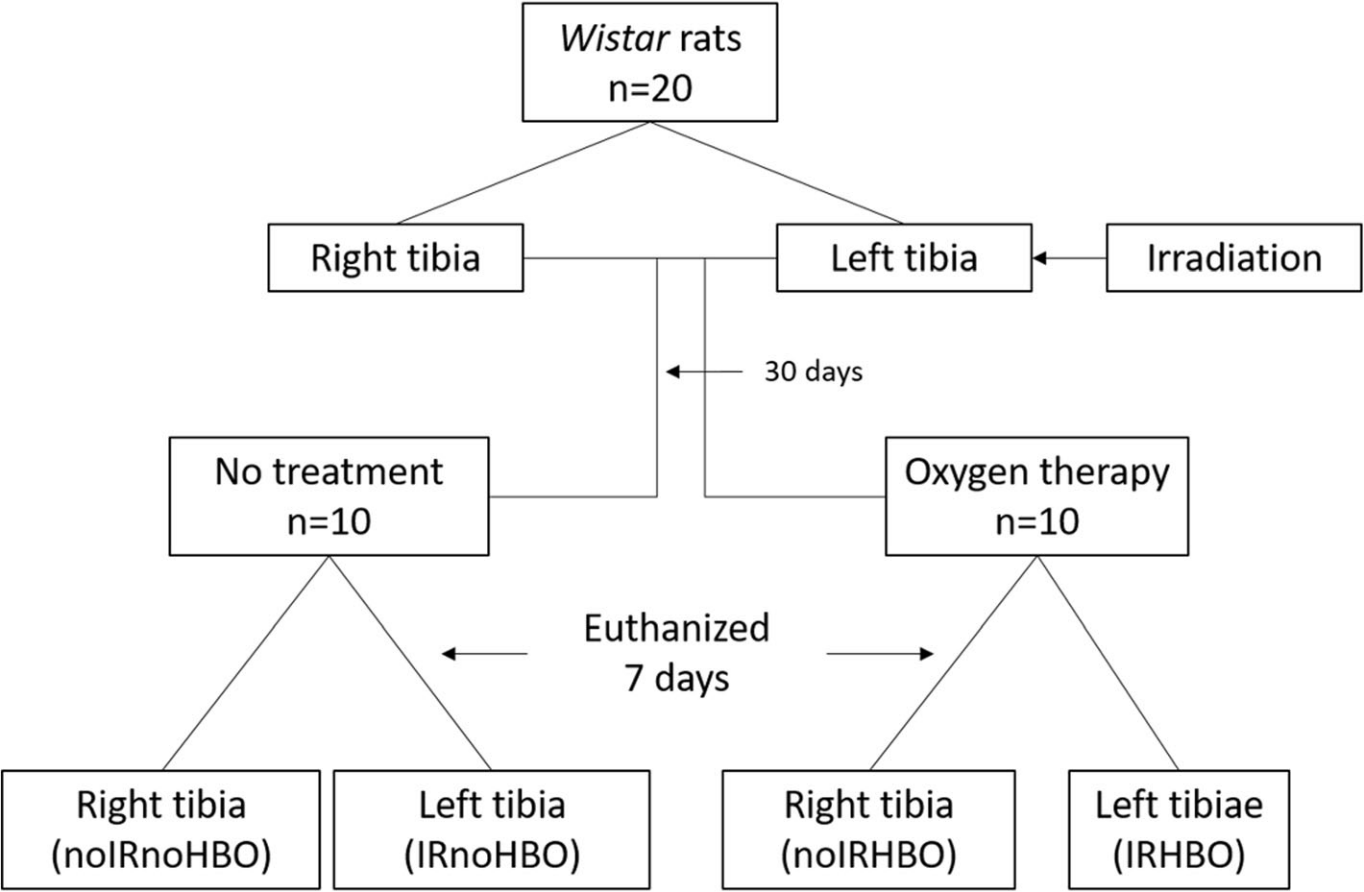
Flow chart summarizing the study design. noIRnoHBO - tibia without irradiation and HBO therapy; IRnoHBO – tibia submitted ionizing radiation, without HBO therapy; noIRHBO – tibia without irradiation and submitted to HBO therapy; IRHBO – tibia submitted to ionizing radiation and HBO therapy

After one week of acclimatization, the animals were anaesthetized by an intraperitoneal injection of 100 mg/kg ketamine 10% and 7 mg/kg xylazine 2% hydrochloride. The left leg was positioned laterally and fixed using a wooden stick and adhesive tape. A 1.5 cm thick wax bolus was positioned over the left tibia and a total dose of 30 Gy was administered in one session, using a linear accelerator (Varian Clinac® 600C S/N 0310, Palo Alto, CA, USA). The left tibiae were designated to the irradiated group and the right tibia that did not receive irradiation, were assigned to the non-irradiated group. The HBO sessions started 30 days after IR in ten animals, and therapies were performed daily for 1 week in a cylindrical pressure chamber (Ecobar 400, Ecotec Equipamentos e Sistemas Ltda®, Mogi das Cruzes, SP, Brazil) at 250 kPa for 90-min sessions after compression (Fig. 1).

The animals were euthanized 37 days after irradiation by intraperitoneal injection with sodium thiopental and lidocaine, followed by cervical dislocation, in compliance with the principles of the Universal Declaration on Animal Welfare. The tibiae were separated into four groups (*n* = 10): from animals without HBO - right tibia Non-irradiated (noIRnoHBO) and left tibia Irradiated (IRnoHBO); and from animals with HBO - right tibiae Non-irradiated (noIRHBO) and left tibia Irradiated (IRHBO). The tibiae were removed by disarticulation, immediately placed in a gauze with physiological saline solution and were stored 2 weeks in freezer storage at − 20 °C. Twenty-four hours before the analysis, the tibiae were defrosted and placed in phosphate buffered saline until analysis.

### Macroscopic analysis

**Fig. 2:**
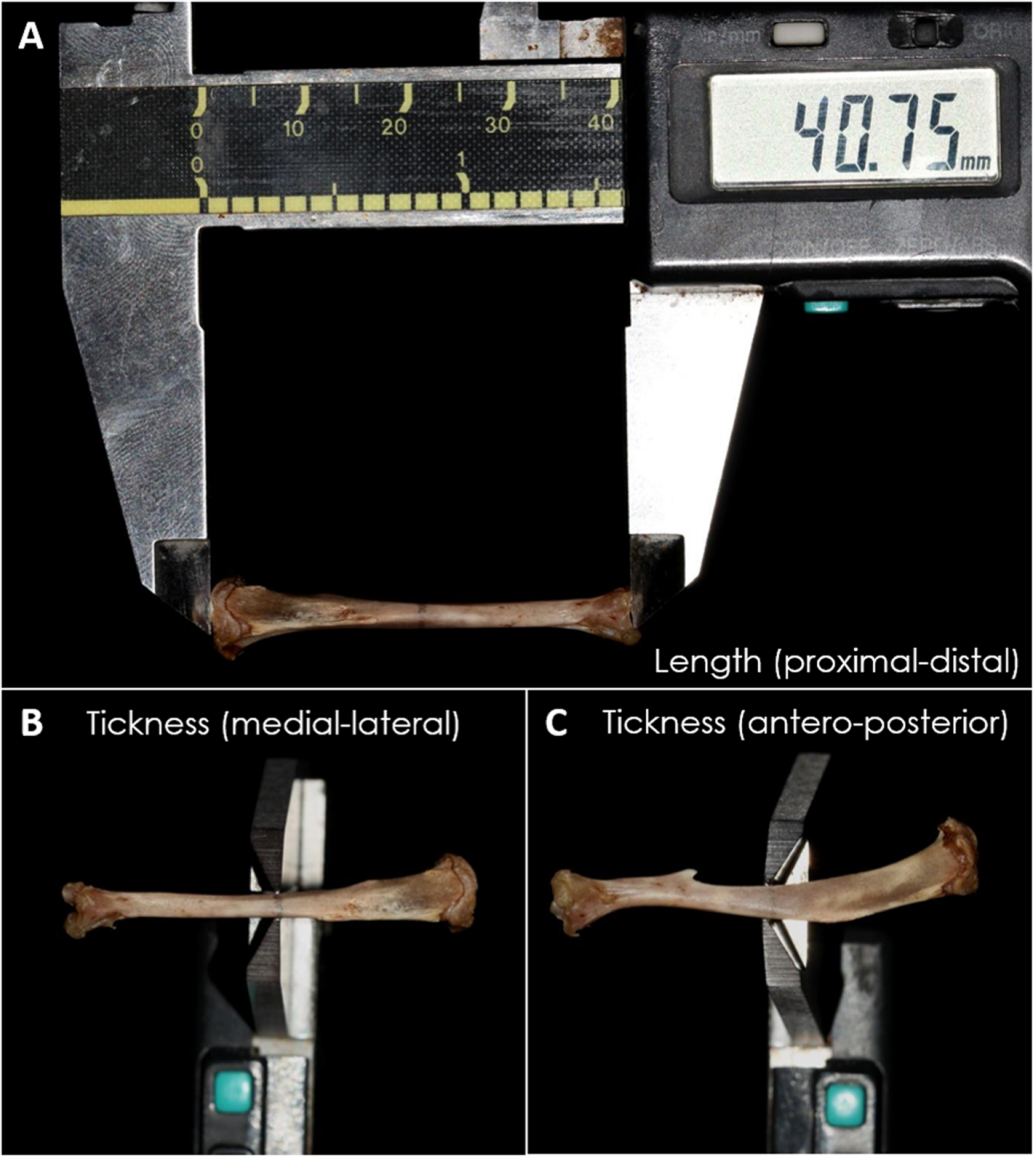
Tibia macroscopic images showing the analysis of length (**a**) and thickness (**b** and **c**) measurement

The tibiae were measured in length - proximal-distal (Fig. 2a), and thickness - medial-lateral and anteroposterior (Fig. 2b, c). The thickness was measured in the middle of the diaphysis. All measurements were taken using a digital pachymeter (Western @PRO DC-6®, São Paulo, Brazil).

### Biomechanics analysis and attenuated Total reflectance (ATR)-Fourier transform infrared spectroscopy (FTIR) analysis

**Fig. 3:**
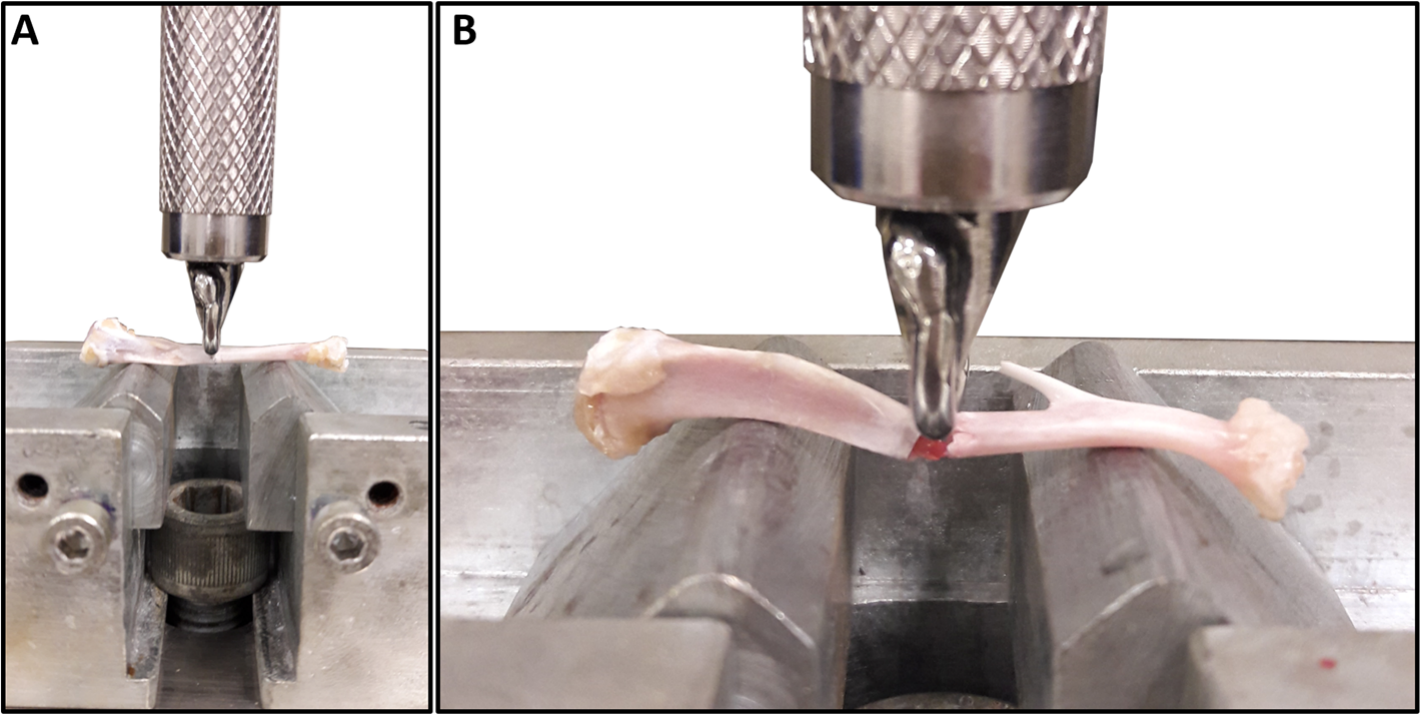
Biomechanical analysis. **a** – The three-bending flexural test, showing the tibia positioned horizontally on the two holding fixtures, with a perpendicular load in middle of bone. **b** – Image showing the moment of bone fracture

After the macroscopy analysis, the tibiae were analyzed in a three-point bending test until failure, using the universal-testing machine (EMIC DL 2000, EMIC Equipamentos e Sistemas de Ensaio Ltda, São José dos Pinhais, Brazil). Each specimen was positioned horizontally on the two holding fixtures with a distance of 16 mm on the machine, while the upper loading fixture applied the force to the middle of the diaphysis at a loading of 20 N at 1.0 mm/min displacement (Fig. 3a). Load and displacement data were recorded and subsequently, load vs. displacement curves were plotted. Evaluations were derived from data with flexural strength (N/mJ) and stiffness values (N/mm). The fractured tibiae (Fig. 3b) were maintained, after the mechanical test, in phosphate buffered saline until the attenuated total reflectance Fourier transform infrared spectroscopy (ATR-FTIR) analysis.

**Fig. 4:**
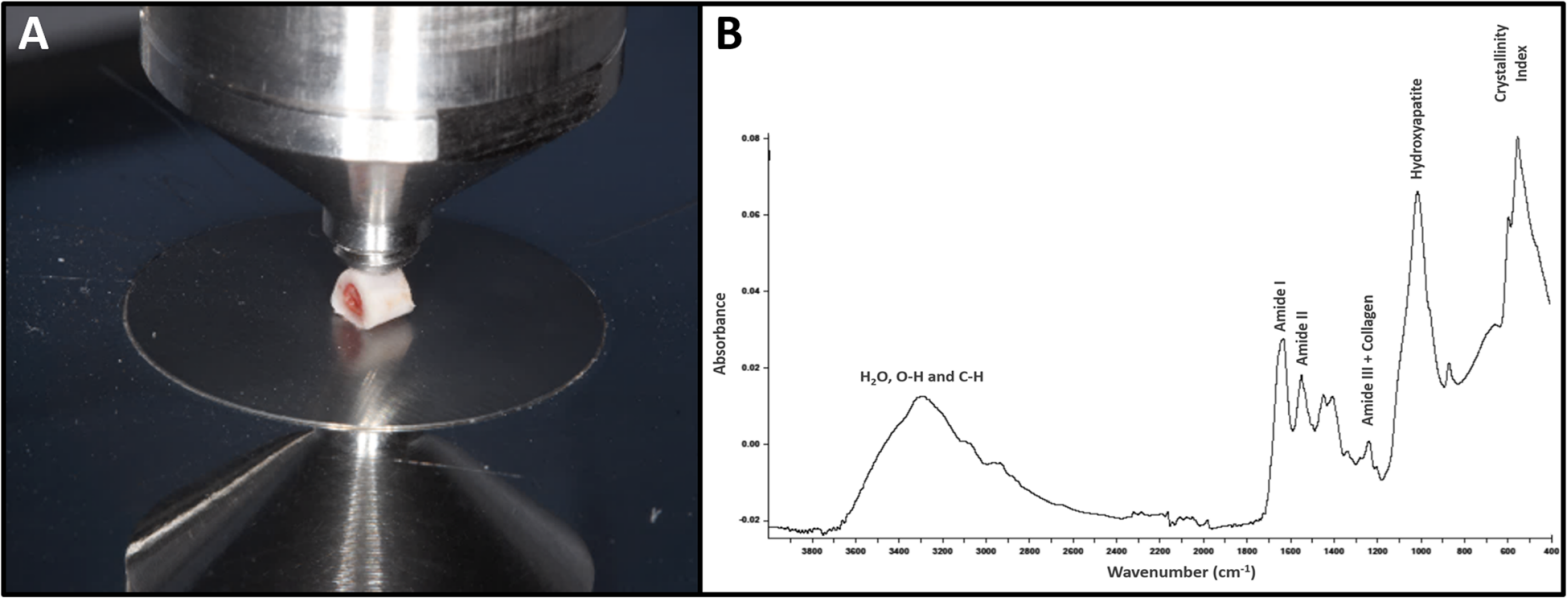
Image of Attenuated Total Reflectance (ATR)-Fourier Transform Infrared Spectroscopy (FTIR) analysis. **a** - The external cortical surface of tibia pressed by a force gauge at ATR-FTIR diamond crystal. **b** – Characteristic bone spectrum obtained from the ATR-FTIR showing the region of the analyzed parameters, using the program OPUS 6.5

The proximal fragment diaphysis was sectioned on the transversal axis with a diamond disk under constant irrigation to obtain three cortical cylindrical fragments. The bone fragments were dehydrated in ovens at 37 °C for one day, and an external cortical surface placed against the diamond crystal of the ATR-FTIR unit, pressed with a force gauge at a constant pressure to facilitate contact (Fig. 4a). Data were recorded and analyzed with OPUS 6.5 software (Bruker®, Ettlingen, Germany). The bone composition was analyzed using Fourier transform infrared spectroscopy (FTIR, Vertex 70 Bruker®, Ettlingen, Germany) equipped with an accessory that allowed for spectrum acquisitions in the attenuated reflectance (ATR) mode. The spectra were recorded in the range of 400 ± 4.000 cm^− 1^ at a 4 cm^− 1^ resolution, and the mean from 32 scans per fragment analyzed was used. Vector normalization and baseline correction were performed across all spectra, and these were considered absorbance height ratios.

The spectra was further analyzed by calculating the following parameters: amide I band (collagen ratio between the mature pyridinoline crosslink peaks (PYR) ± 1660 ^cm-1^ and the immature crosslinking dihydroxynorleucine (DHLNL) - 1690 cm^− 1^); crystallinity Index (the intensity ratio of peaks 551 and 597 cm^− 1^ for 588 cm^− 1^); and matrix-to-mineral ratios of amide I + II/hydroxyapatite (HA) (M:MI) (the ratio between the integrated areas of amide I + II (1520 ± 1720 cm^− 1^) for HA (916 ± 1180 cm^− 1^)) and amide III + collagen/HA (M:MIII) (the ratio between the integrated areas of amide III (1210 ± 1270 cm^− 1^) with two collagen bands (1269 ± 1296 cm^− 1^ and 1180 ± 1213 cm^− 1^) for HA (916 ± 1180 cm^− 1^) (Fig. 4b).

### Statistical analysis

The data from all measured parameters were tested for normal distribution (Shapiro-Wilk) and the equality of variances (Levene’s test). The data submitted to the normality and equality of variance tests showed a parametric distribution and the results were expressed in mean and standard deviation. Thereby, the parametric test Two-way analysis of variance (ANOVA) was performed followed by the Tukey test. All tests employed a level of significance of α = 0.05 and all-statistical analyses were carried out with Sigma Plot version 13.1 (Systat Software Inc., San Jose, CA, USA).

## Results

The means and standard deviations of all parameter analyses are shown on Table 1. In the macroscopy analysis, the IRnoHBO and IRHBO groups showed lower length values compared to noIRnoHBO and noIRHBO groups (*p* < 0.01). The thickness of the anteroposterior (AP) and medial-lateral (ML) parameters showed lower values in IRnoHBO and IRHBO compared to noIRnoHBO and noIRHBO groups, respectively (AP: *p* < 0.03 ML: *p* < 0.02). In addition, the IRnoHBO has no statistical difference in macroscopic analysis compared to IRHBO (*p* > 0.05).

**Table 1:** The means and standard deviation values of all parameters analysis

Tests/Groups	noIRnoHBO	noIRHBO	IRnoHBO	IRHBO
**Length (proximal-distal)**	37.67 ± 1.59	37.00 ± 0.95	35.30 ± 1.36	34.80 ± 1.22
	Aa	Aa	Ba	Ba
**Thickness (anteroposterior)**	3.00 ± 0.15	2.91 ± 0.30	2.81 ± 0.22	2.76 ± 0.23
	Aa	Aa	Ba	Ba
**Thickness** **(medial-lateral)**	2.29 ± 0.16	2.21 ± 0.18	2.15 ± 0.17	2.09 ± 0.14
	Aa	Aa	Ba	Ba
**Flexural strength**	58.60 ± 14.69	73.37 ± 23.86	45.66 ± 18.30	59.66 ± 19.46
	Ab	Aa	Bc	Bb
**Stiffness**	129.10 ± 19.45	120.75 ± 8.97	89.06 ± 13.96	106.00 ± 13.95
	Aa	Aa	Bb	Aa
**Amide I ratio**	2.56 ± 0.29	2.41 ± 0.44	2.04 ± 0.44	1.82 ± 0.34
	Aa	Aa	Bb	Bb
**Crystallinity Index**	2.96 ± 0.27	3.09 ± 0.41	2.65 ± 0.42	2.80 ± 0.31
	Aa	Aa	Bb	Bb
**Amide I + II/ Hydroxyapatite**	0.43 ± 0.14	0.36 ± 0.10	0.34 ± 0.06	0.29 ± 0.08
	Aa	Aa	Bb	Bb
**Amide III + Collagen/ Hydroxyapatite**	3.57 ± 1.31	4.75 ± 1.31	1.93 ± 0.42	1.53 ± 0.31
	Aa	Aa	Bb	Bb

Groups: Non-irradiated (noIRnoHBO) and Irradiated (IRnoHBO) - animals without HBO; and Non-irradiated (noIRHBO) and Irradiated (IRHBO) - animals’ treatment with HBO. Different upper case letters within rows indicate significant differences for systemic condition factor (non-irradiated or irradiated); different lower case letters within rows indicate significant difference for HBO therapy factor (non-HBO or HBO therapy). Comparison performed by Tukey test (*p* < 0.05)

The biomechanics analysis showed that the IRnoHBO group had lower value of flexural strength, when compared to the noIRnoHBO and IRHBO groups (*p* < 0.04). In addition, the noIRHBO group showed a higher value of flexural strength compared to noIRnoHBO and IRHBO groups (*p* < 0.02). The stiffness parameter showed that IRnoHBO had lower values compared to noIRnoHBO and IRHBO groups (p < 0.03), however, there is no statistical difference in noIRHBO, when compared to the IRHBO and noIRHBO groups (*p* > 0.06).

In the ATR-FTIR analysis, the IRnoHBO and IRHBO had lower values of collagen maturity, when compared to the noIRnoHBO and noIRHBO groups, respectively (p < 0.03). The crystallinity index showed that IRnoHBO and IRHBO had lower values, when compared to the noIRnoHBO and noIRHBO groups, respectively (p < 0.04). In addition, the organic/inorganic ratios (M:MI and M:MIII) showed that IRnoHBO and IRHBO had lower values compared to the noIRnoHBO and noIRHBO groups (M:MI: *p* < 0.01; M:MIII: p < 0.02).

## Discussion

The present study showed that IR compromises bone growth, decreases mature/immature crosslinks ratio, changes morphology of HA crystals and collagen/HA ratio, decreasing flexural strength and stiffness in rat tibiae. HBO therapy improves flexural strength and stiffness parameters in irradiated tibia, showing no statistical difference with the noIRnoHBO group.

The 30 Gy used was based on previous studies, which showed that a single high dose of IR was similar to 50–70 Gy fractional radiotherapy received in most patients with carcinoma [15, 16]. Studies have shown that these doses can arrest cell cycle progression, allowing evaluations of the irradiation effects on bone tissue [7, 17]. HBO therapy has been widely used in situations where irradiation compromised microcirculation [2, 3], and the protocol accepted in animal studies involves the delivery of 100% oxygen at 150 to 300 kPa for 60 to 90 min, once daily [10, 11], as used in the present study. In addition, the period of 30 days after ionizing radiation is the time required for structural changes in bone tissue [7]; and the 7-day period of HBO therapy was used to evaluate the initial treatment response in bone compromised by IR.

Our results showed that in the macroscopy analysis, the irradiated groups (IRnoHBO and IRHBO) had lower length and thickness (anteroposterior and medial-lateral) values when compared to the non-irradiated groups (noIRnoHBO and noIRHBO). Macroscopic changes, such as growth arrest and/or angular deformity of the extremity or kyphosis and spine scoliosis, are frequently reported when the irradiation field includes the growth plate [5, 18]. IR compromises the endochondral ossification process through impairment of chondrocytes proliferation [19] in the serial cartilage zone. Moreover, IR damages small blood vessels in the ossification zone that blocks osteogenesis, thus preventing normal remodeling at the chondroosseous junction [12]. In addition, noIRHBO and IRHBO showed no significant difference in macroscopic analysis compared to noIRnoHBO and IRnoHBO, respectively. This suggests that irradiation, applied during the animal growth, significantly damages and impairs the ossification process [5]. In addition, HBO therapy did not show significant improvement, after the bone was compromised.

In the FTIR analyses, our results showed that IRnoHBO and IRHBO had lower values of collagen maturity, crystallinity index and organic/inorganic ratios, when compared to the noIRnoHBO and noIRHBO groups. Studies have shown that irradiation induces side chain decarboxylation of the collagen molecule, thus modifying the interaction or binding between the organic matrix and the HA mineral [6, 20]. This increases immature cross-links on collagen [21], changes the morphology HA crystals [22] and impairs the mineralization process [7]. The present study showed that the noIRHBO and IRHBO groups demonstrated no significant difference for the ATR-FTIR analysis, when compared to the noIRnoHBO and IRnoHBO groups, respectively. This methodology analyzes nanostructure changes, then our results suggest that the HBO therapy did not minimize the IR deleterious effects, however, some microstructural changes might have occurred, according to the findings in the biomechanical analysis.

In the biomechanics analysis, the IR groups showed lower values of flexural strength and bone stiffness, leading to greater susceptibility to fractures. The collagen arrangement and the interaction with apatite crystals are important for establishing mechanical and structural properties of bone [23]. The primary aspect of the irradiation-induced loss of fracture resistance could be due to the complete loss of plastic deformation (intrinsic toughness) after irradiation [20], induced by damaging collagen molecules [21]. In the present study, the IRHBO group showed no statistical difference of flexural strength and stiffness, when compared to the noIRnoHBO group. The study showed that HBO therapy holds the potential to increase intermolecular interactions (by hydrogen bonds) in the collagen, followed by induced cross-linking that stabilizes the fibrils [9]. Our study suggests that IR decreases the number of intermolecular interactions in collagen molecules and HBO therapy improves the quality of these remaining interactions. This is in agreement with previous reports that HBO therapy increases bone mechanical properties by increasing the organization of collagen fibers [24, 25]. However, further studies are required to clarify the molecular mechanisms underlying intermolecular interaction under HBO therapy conditions.

The morphologic and biomechanical alterations in bone induced by a high dose of IR are a major concern for surgeons who are considering rehabilitation in patients after therapeutic irradiation treatment. Studies suggests that, for people with irradiation tissue injury, HBO therapy is associated with an improved outcome, including cases with severe mandibular osteoradionecrosis [2, 3, 26]. However, other studies showed controversy regarding the clinical effectiveness of HBO therapy in bones compromised by radiotherapy [27–29]. Although, these studies have some bias, such as variations in radiation dosage, patients excluded on the basis of advanced osteoradionecrosis, HBO protocol, adjunctive therapy other than HBO, time between radiation and tooth extraction, method of extraction, and adjunctive therapy other than radiation. Although the results of the present animal study cannot be extrapolated to humans [30], but, serve to support new research in this area.

## Conclusion

The results of the present study showed that ionizing radiation arrests bone development, as well as decreasing collagen maturation and the mineral deposition process, along with reducing the flexural strength and bone stiffness mechanical parameters. Moreover, HBO therapy was shown to minimize deleterious effects of irradiation on flexural strength and the bone stiffness analysis.

## Data Availability

The datasets used and/or analyzed during the current study are available from the corresponding author on reasonable request.

## Abbreviations

IR: Ionizing radiation
HAP: Hydroxyapatite
HBO: Hyperbaric Oxygen
kPa: Kilopascal
ATR-FTIR: Attenuated Total Reflectance Fourier Transform Infrared Spectroscopy
PYR: Pyridinoline
DHLNL: Dihydroxynorleucine
AI: Amide I band
CI: Crystallinity Index
M:MI: Matrix-to-mineral ratio: Amide I + II/Hydroxyapatite
M:MIII: Amide III + Collagen/HA
